# Design and Characterization of Thermosensitive Niosomes as Platforms for Daunorubicin Delivery

**DOI:** 10.3390/ph18091375

**Published:** 2025-09-15

**Authors:** Viliana Gugleva, Katerina Ahchiyska, Elena Drakalska-Sersemova, Rositsa Mihaylova, Natalia Toncheva-Moncheva, Erik Dimitrov, Krum Aleksandrov, Aleksander Forys, Barbara Trzebicka, Denitsa Momekova

**Affiliations:** 1 Department of Pharmaceutical Technologies, Faculty of Pharmacy, Medical University of Varna, 84 Tsar Osvoboditel Str., 9000 Varna, Bulgaria; viliana.gugleva@mu-varna.bg; 2 Department of Pharmaceutical Technology and Biopharmaceutics, Faculty of Pharmacy, Medical University of Sofia, 2 Dunav Str., 1000 Sofia, Bulgaria; katerina.ahchiiska@gmail.com; 3 Faculty of Medical Sciences, Goce Delcev University, 2000 Stip, North Macedonia; elena.drakalska@ugd.edu.mk; 4 Department of Pharmacology, Pharmacotherapy and Toxicology, Faculty of Pharmacy, Medical University of Sofia, 2 Dunav Str., 1000 Sofia, Bulgaria; rmihaylova@pharmfac.mu-sofia.bg; 5 Institute of Polymers, Bulgarian Academy of Sciences, bl.103 Akad. G. Bonchev Str., 1113 Sofia, Bulgaria; ntoncheva@polymer.bas.bg (N.T.-M.); e_dimitrov@polymer.bas.bg (E.D.); k_aleksandrov@polymer.bas.bg (K.A.); 6 Centre of Polymer and Carbon Materials, Polish Academy of Sciences, ul. M. Curie-Skłodowskiej 34, 41-819 Zabrze, Poland; aforys@cmpw-pan.pl (A.F.); btrzebicka@cmpw-pan.pl (B.T.)

**Keywords:** antiproliferative activity, carboxyfluorescein, daunorubicin hydrochloride, cancer, niosomes, thermosensitivity

## Abstract

**Background/Objectives:** The study describes the elaboration and evaluation of thermosensitive niosomes intended for the systemic application of daunorubicin hydrochloride. The attained stimulus sensitivity would determine the release of the chemotherapeutic predominantly at the target site, which ensures a higher drug concentration and leads to reduced systemic toxicity. The latter is highly beneficial, as the anthracycline antibiotic is known for its dose-dependent cardiotoxic effects. **Methods**: Conventional and copolymer-modified niosomes were prepared via thin-film hydration and the transmembrane ammonium gradient method, allowing us to assess the impacts of copolymer type-DHP-PiPOX (1,3-dihexadecyl-propane-2-ol-poly(2-isopropyl-2-oxazoline)) or DHP-PETEGA (1,3-dihexadecyl-propane-2-ol-poly(ethoxytriethylene glycol acrylate)) and their concentrations (0.5, 1, and 2.5 mol%), as well as the method of preparation, on the main physicochemical properties of the vesicles. Niosomes were characterized in terms of their size, polydispersity index (PDI), zeta potential, entrapment efficiency, morphology, and drug release properties. Thermosensitivity was evaluated by fluorescence studies, and the antiproliferative activity of optimized formulations was assessed against the acute myelocyte leukemia-derived HL-60 cell line. **Results**: Daunorubicin-loaded niosomes modified with DHP-PiPOX and DHP-PETEGA at 2.5 mol% exhibited suitable physicochemical properties for systemic application, with sizes below 200 nm (155 and 158 nm respectively), low PDI values of 0.25 and 0.29, spherical morphology, and high daunorubicin entrapment efficiency (68.6 and 66.5% respectively). The vesicles showed temperature-dependent drug release properties and superior antiproliferative activity compared to the free daunorubicin (IC_50_ values of 6.91 and 8.54 vs. 12.14). **Conclusions**: The obtained results indicate that the developed thermosensitive nanovesicles may serve as a suitable drug delivery system for the systemic application of daunorubicin hydrochloride.

## 1. Introduction

In the recent years, multifunctional or “smart” nanocarriers have attracted researchers’ attention due to the numerous beneficial characteristics they provide compared to conventional nanoscale drug delivery systems. The selected trigger/s allow for controlled and precise drug release at the desired site, which contributes to a superior therapeutic effect and reduced systemic toxicity. Stimuli-sensitive nanocarriers are often elaborated as drug delivery platforms in oncotherapeutics, as the altered tumor microenvironment (e.g., pH 5.5–6.5, mild hyperthermia at 40–42 °C, a higher glutathione concentration, or overexpression of certain enzymes) provides a variety of trigger mechanisms that can be exploited [1]. This approach for targeted delivery is often considered to be more specific compared to the classical active or passive strategies. However, the success of the latter may be limited by the heterogeneous expression of receptors on the target site; the strong bonds formed between the receptor and ligand, hindering the penetration of the loaded drug; or the higher propensity for opsonization of the functionalized vesicles in the systemic circulation [2].

Niosomes, non-ionic surfactant-based vesicles that are analogous to liposomes, have gained significant attention as carriers for anticancer drugs due to their biocompatibility, stability, and capacity for both hydrophilic and hydrophobic payloads [3]. However, conventional niosomes often exhibit insufficient control over drug release and lack precise site-specific delivery, especially in the context of solid tumors, where enhanced permeability and retention (EPR) effects are variable and inconsistent [4].

To overcome these limitations, the integration of stimuli-responsive features into niosomal systems has emerged as a promising approach. Thermosensitive niosomes, which respond to hyperthermic conditions (typically 40–42 °C), offer a spatiotemporally controllable mechanism for drug release by exploiting the elevated-temperature microenvironment of tumors or externally induced hyperthermia [5]. Thermosensitive niosomes are elaborated by incorporating thermoresponsive segments such as surfactants, lipids, or polymers, which can undergo a phase transition following a temperature gradient [5]. In their study, Tavano et al. prepared thermosensitive calcein/5-fluorouracil-loaded niosomes based on L64 Pluronic^®^ and the derivative L64ox with or without cholesterol [6]. Their in vitro release studies conducted at different temperatures—25, 37, and 42 °C—revealed that niosomes based on L64 Pluronic (without cholesterol) possess thermosensitive properties—the highest release rate was observed at 42 °C, irrespective of the type of loaded cargo [6]. In another study, as a thermosensitive ligand, the researchers used dipalmitoyl phosphocholine incorporated into PEGylated niosomes loaded with CDC20siRNA, doxorubicin, and quercetin. The prepared vesicles underwent a phase transition at a temperature of 40 °C and released the encapsulated drugs. The positive charge of niosomes determined their high degree of cellular uptake following the electrostatic interactions that occurred with biological membranes [7].

In this context, the present study explores the development of polymer-modified thermosensitive niosomes as a site-specific nanovesicular system for daunorubicin delivery. Thermosensitivity was achieved using copolymers newly synthesized for this purpose, namely DHP-PiPOX and DHP-PETEGA, which implies the novelty of our work, as currently there are no data in the literature from relevant studies. The designed niosomes integrate three key functional features: (i) thermal sensitivity, as a result of the low critical transition temperature of PIPOX or PETEGA blocks enabling temperature-triggered changes in niosomal membrane fluidity and permeability, which can facilitate controlled release of encapsulated drugs at mild hyperthermic tumor sites or inflamed tissues; (ii) hydrophilic poly(ethoxytriethylene glycol acrylate) or polyoxazoline form a stealth-like corona that reduces opsonization and prolongs circulation time; and (iii) encapsulation of daunorubicin for targeted chemotherapy. Regarding the selection of the active pharmaceutical ingredient (API) in our study, daunorubicin HCl was chosen as it is a broad-spectrum cytostatic agent of importance in the treatment of a number of neoplastic diseases [8]. It is used as first-line induction therapy in cases of acute myeloid leukemia (AML) in pediatric patients. Approximately 80–90% of treated patients achieve complete remission after completing the therapy, with a 5-year survival rate of 60–70%. For older patients (<60 years), the successful rates are lower—approx. a 60–80% remission rate and a 40–50% 5-year survival rate are achieved [9]. Daunorubicin is characterized by complex pharmacokinetics, with a three-compartment distribution model with three-phase elimination, and there is significant accumulation in tissues and organs with a rich blood supply, such as the heart, bone marrow, liver, etc. [10]. These pharmacokinetic features are a prerequisite for the significant adverse and toxic effects of daunorubicin. Like other anthracyclines, the main dose-limiting toxicity of this agent is cardiotoxicity, manifested clinically as cardiomyopathy and digitalis glycoside-refractory congestive heart failure [11,12]. The risk of cardiotoxic effects increases significantly after reaching a cumulative dose of over 1000 mg/m^2^. Its unfavorable toxicological profile has led to great interest in its development in nanovesicular carriers, which provide optimization of efficacy while simultaneously reducing systemic exposure and dose-limiting side effects and toxic effects. Currently, there are two nanoformulations containing daunorubicin that are implemented in clinical practice—the liposomal DaunoXome^®^, intended for treatment of Kaposi’s sarcoma related to HIV/AIDS, and Vyxeos^®^ (liposomal daunorubicin and cytarabine), used for the therapy of AML. Regarding current research progress, daunorubicin HCl was reportedly loaded into the structure of niosomes, as well as into thermosensitive liposomes [13]. Balasubramaniam et al. (2002) prepared conventional niosomes loaded with the anthracycline antibiotic characterized by prolonged release and an improved antitumor effect [14], whereas Liu et al. (2017) developed anti-CD123 antibody-functionalized niosomes for targeted delivery of the chemotherapeutic [15]. In their study, Alrbyawi developed thermoresponsive liposomes dual-loaded with daunorubicin and cardiolipin, which were characterized by rapid drug release at 42 °C and fourfold-higher cytotoxic activity compared to the plain drug and PEGylated vesicles [16]. Regarding the clinical significance of thermosensitive nanocarriers in practice, the thermoresponsive liposomal doxorubicin, ThermoDox^®^, is noteworthy, as it has been reported to suppress tumor growth at temperatures above 39.5 °C [17]. The biocompatibility and stability of niosomes make them an attractive alternative to conventional nanovesicular formulations such as liposomes, further supporting their potential in advancing cancer therapeutics. Furthermore, the integration of temperature sensitivity into niosomes represents a significant step towards optimized targeted delivery of daunorubicin.

In this regard, the aim of the study is to develop and characterize thermosensitive niosomes that are capable of selectively releasing daunorubicin HCl in conditions of mild hyperthermia (42 °C) and to evaluate its antiproliferative activity against acute myelocyte leukemia-derived HL-60 cell lines. The controlled temperature-triggered drug release would contribute towards achieving a higher drug concentration, reduced systemic exposure, and an improved therapeutic effect.

## 2. Results and Discussion

### 2.1. Preparation and Characterization of Conventional and Copolymer-Modified Blank Niosomes—Preliminary Studies

The objective of this study was to design temperature-sensitive niosomal nanovesicles for targeted daunorubicin release and delivery. These innovative nanocarriers can enhance the delivery of daunorubicin, a potent anticancer drug, directly to tumor cells, thereby minimizing systemic toxicity and side effects on healthy tissues. They offer a controlled release mechanism triggered by temperature changes, allowing precise drug accumulation at specific sites within the body.

To this end, a series of Tw60:Sp60:Ch (3.5:3.5:3 mol:mol) niosomes modified with DHP-PiPOX or DHP-PETEGA copolymers were prepared via the TFH method to evaluate the influence of the type and concentration of the modified copolymer on the main physicochemical properties of the niosomes, in accordance with their specific temperature trigger (Table 1). The composition of the niosomal membrane was chosen based on previous studies proving that this mixture of surfactant and cholesterol is optimal for loading both hydrophilic and hydrophobic drugs [18]. In their study, Junyaprasert et al. (2012) [19] also exploited the combination of both surfactants as noisome-forming components in addition to various solubilizers (polyethylene glycol 400, propylene glycol, methanol) in order to encapsulate ellagic acid. The optimal formulation based on Span 60:Tween 60 (2:1) exhibited suitable physicochemical properties for dermal delivery and superior percutaneous permeation compared to the plain solution.

Although the formation of conventional niosomes based on non-ionic surfactant and cholesterol mixtures has been extensively studied, research on the preparation of stimuli-sensitive polymer-modified niosomes so far remains scarce in the literature. To the best of our knowledge, this is the first report on the preparation of temperature-sensitive niosomes by grafting temperature-responsive polymers such as PiPOX and PETEGA to their membranes. Consequently, it was necessary to confirm the formation of niosomes through cryo-TEM analysis.

**Figure 1:**
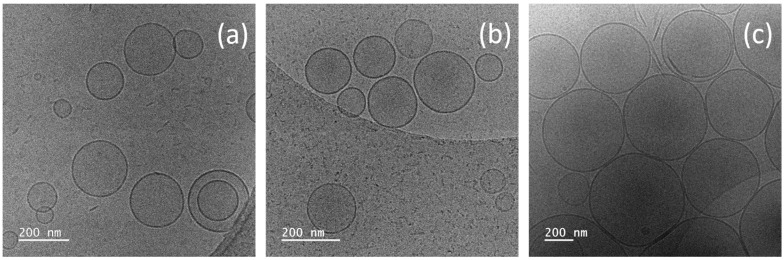
Cryo-TEM micrographs of plain (**a**) and temperature-responsive niosomes modified with 2.5 mol% DHP-PiPOX (**b**) and DHP-PETEGA (**c**).

The obtained cryo-TEM images of non-sonicated plain and modified niosomes show the formation of well-defined spherical vesicles with intact membranes of approx. 5 nm thickness and an average size (based on observation of at least 100 particles per sample) varying in the range of 263 to 545 nm, depending on the type and concentration of copolymer used. Additionally, within the series of modified niosomes, the incorporation of the two hydrophobically modified polymers at concentrations up to 2.5 mol% did not compromise the niosomal bilayer structure. The images were dominated by unilamellar vesicles (Figure 1). Only in a low number of images were small fractions of oligolamellar niosomes and rod-like structures observed.

Similarly to cryo-TEM, the DLS analysis data of the obtained niosomes show sizes over 250 nm and a polydispersity index over 0.4, which are unfavorable characteristics in view of the potential systemic application of the developed niosomes. This necessitated, as the next stage of the optimization process, their treatment with ultrasound for 2 min (20 on/10 off s). The results are shown in Table 1.

As evident from the obtained data, the inclusion of the polymers in a concentration up to 2.5 mol% determines the formation of homogeneous populations of nanocarriers with sizes between 100 and 133 nm at ambient temperature and a relatively low dispersity index of up to 0.36. The incorporation of the modifying polymers leads to a statistically significant (*p* < 0.05) decrease in size in comparison with plain niosomes, which may be related to the interactions between the hydrophobic anchor of the polymers (DHP) and non-ionic surfactants’ molecules and compaction of the bilayer. Similar findings were reported by other authors after the encapsulation of hydrophobic drugs in niosomal membranes [20]. On the other hand, within the series of copolymer-modified vesicles, a clear trend of a concentration-dependent increase in the size of the vesicles was observed. Among the studied polymer concentrations, the largest sizes were invariably reported at 2.5 mol%, probably due to the more pronounced steric repulsion between the hydrophilic polymer chains above the membrane surface, causing their expansion and transition from mushroom to brush conformation, followed by a corresponding increase in niosomal size. Regarding zeta potential values, the inclusion of the modifying copolymers was associated with decrease in zeta potential compared to plain vesicles, resulting in more negative values ranging from −10.7 to −44 mV, which are considered suitable for providing colloidal stability in the dispersions.

The thermosensitivity of the niosomes was assessed by incubating the vesicles for 40 min at 40 °C, the recommended conditions for mild hyperthermia in cancer treatment [5,21]. This elevated temperature led to an increase in vesicle size, except for in the case of S5, which may be explained by changes in solubility of the hydrophilic blocks (PiPOX and PETEGA) depending on the temperature [22]. At 40 °C, the solubility of both polymers decreased as they approached their cloud point temperature, where a transition from hydrophilic to more hydrophobic behavior occurs (Table 2). This transition likely promotes the formation of rigid structures from the PIPOX or PETEGA chains around the niosomal membrane, leading to the observed increase in vesicle dimensions and highlighting the temperature-dependent behavior of the systems [21]. Temperature dependence was also observed in the change in zeta potential values, especially in the series of niosomes modified with DHP-PIPOX, where a decrease in the absolute value of this parameter was recorded. The observed changes are probably due to disorganization in the conformation or spatial orientation of the hydrophilic PIPOX chains and, consequently, disorganization of the niosomal membranes [6,23].

**Table 2:** Cloud points and thermal (DSC) characterization of PIPOX and PETEGA.

Sample Code	Cloud Point (°C)	T_g_^c^ (°C)
PiPOX [24]	39	52
PETEGA [25]	37	−57

^c^ Glass transition temperature from second heating run.

**Figure 2:**
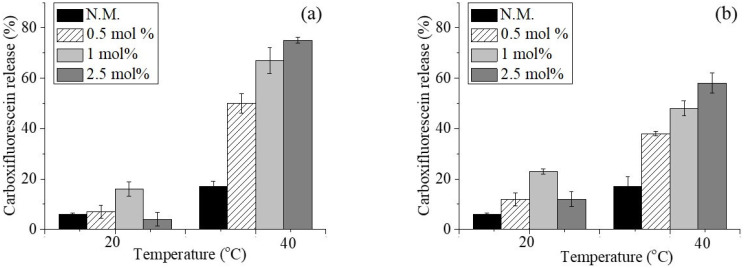
Carboxyfluorescin release from DHP-PiPOX-modified (**a**) and DHP-PETEGA-modified (**b**) niosomes after 40 min of incubation at 20 and 40 °C in phosphate buffer medium at pH 7.4.

The successive stage of our study was to assess the acquired stimulus sensitivity of the vesicles by conducting a leakage assay. The fluorescent dye 5(6)-carboxyfluorescein, polar and non-permeable through the intact membranes, was loaded into niosomes at a concentration of 50 µM, at which concentration its fluorescence is self-quenching. Upon its leakage into the acceptor medium through pores or other imperfections in membrane integrity caused by polymer incorporation, the dye is diluted and begins to fluoresce. The results from the performed spectrofluorometric studies are presented in Figure 2.

As the presented data show, modification of niosomes with the newly synthesized polymers led to the rapid release of the fluorescent dye within 40 min of incubation at 40 °C. The most pronounced effect was seen in formulations modified with the highest concentration (2.5 mol%) of both polymers, especially those modified with DHP-PIPOX, where almost 80% of the encapsulated carboxyfluorescein was released. Conversely, at ambient temperature, DHP-PIPOX exhibited a stabilizing effect on the membranes, particularly at 2.5 mol%, as evidenced by the lowest carboxyfluorescein leakage compared to the plain vesicles or their DHP-PETEGA-modified counterparts. An anomalous variation in this trend was observed for formulations modified with 1 mol% of both polymers (S3 and S6), where an increase in leakage of the fluorescent marker was observed at ambient temperature. One possible explanation is the initiation of conformational changes in the PIPOX and PETEGA chains above the niosomal membrane at this particular concentration, leading to the formation of transient pores or openings in the bilayers [26]. Further increasing the copolymer concentration to 2.5 mol% led to pronounced stabilization of the vesicles. Thus, the attained thermosensitivity, together with the steric stability of the polymer-modified niosomes, may serve as a feasible approach for enabling targeted drug release within the tumor interstitium.

On this ground, the next step in our study was the elaboration of temperature-sensitive niosomes for targeted delivery of daunorubicin. As an optimal formulation for this purpose, we chose ones based on a 2.5 mol% copolymer content.

### 2.2. Preparation and Characterization of Temperature-Sensitive Daunorubicin Hydrochloride-Loaded Niosomes

Pilot experiments were conducted to establish a suitable and reproducible method for the optimal loading of daunorubicin.HCl into niosomes. These experiments used passive loading via the simple thin-film hydration method and active loading by generating a transmembrane ammonium gradient. Plain and 2.5 mol% Tw60:Sp60:Chol (3.5:3.5:3 mol:mol)-modified niosomes were used as carriers in all experiments. The vesicles were prepared as described in Section 3.2.1, and the amount of encapsulated drug was determined using a spectrophotometric method previously validated as specified in Section 3.3.3. The encapsulation efficacy data are presented in Table 3.

As expected, passive loading did not result in efficient encapsulation. Less than 20% of the daunorubicin was encapsulated in the vesicles. However, after active loading via a transmembrane ammonium gradient, the encapsulation efficiency increased significantly to 71% (Table 3). The incorporation of thermosensitive copolymers had only a marginal effect on the encapsulation efficacy.

DLS analysis of the loaded vesicles revealed that encapsulation of daunorubicin hydrochloride into niosomes led to a significant increase in the vesicles’ size as compared to their empty counterparts (Table 4), but still, their dimensions remained suitable for potential systemic application. This may be due to the interaction of amphiphilic daunorubicin molecules with surfactants on the niosomal membranes, leading to increased repulsion of the bilayers, and hence an increased vesicle size [27]. Regardless the increase size, all loaded formulations are characterized with monomodal size distribution and low polydispersity indices (Table 4). Only small changes towards less negative zeta potential values of the loaded vesicles after daunorubicin encapsulation were observed.

### 2.3. In Vitro Daunorubicin Release

A primary goal in the design of smart nanocarriers is to selectively accumulate the loaded drug in foci of malignant growth, while reducing systemic exposure. This can only be achieved if the nanovesicles effectively retain their cargo in circulation and release it upon accumulation in the tumor interstitium [28].

**Figure 3:**
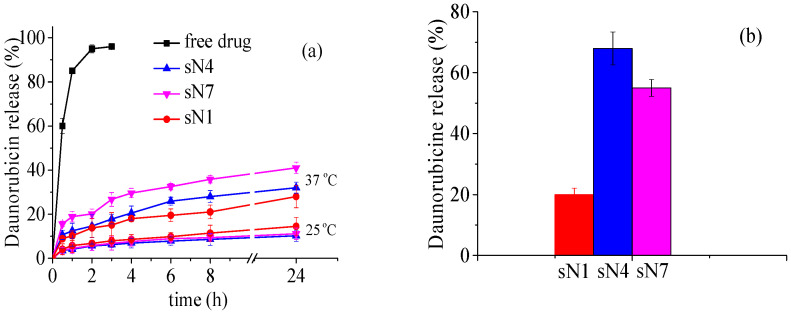
Daunorubicin HCl release from niosomes as a function of time at 25 °C and 37 °C (**a**) and at 42 °C for 40 min (**b**).

In this regard, the release profiles of daunorubicin hydrochloride from the prepared niosomal formulations were studied by dialysis against a 50 mL phosphate-buffered saline (PBS) solution (pH 7.4), as a function of time and temperature at 25, 37, and 42 °C (Figure 3a,b).

At ambient temperature, both unmodified and specially polymer-modified thermosensitive formulations exhibited a slow release rate, with around 10% of the encapsulated daunorubicin HCl released after 24 h (see Figure 3a). Increasing the temperature to physiological values (37 °C) resulted in higher release rates, particularly for the DHP-PETEGA formulation (41% of the drug was released), and rates of 28% and 32% were observed for the unmodified and DHP-PiPOX-modified vesicles, respectively. The faster release observed from PETEGA-modified carriers is probably due to the lower critical solution temperature (LCST) of this copolymer (Table 2); changes in membrane integrity begin at physiological temperature as a result. However, after 24 h of incubation, both the unmodified and thermosensitive niosomes retained over 60% of the loaded cytostatic agent, which is a prerequisite for reduced systemic toxicity.

Daunorubicin release from the developed vesicles was also monitored at 42 °C after 40 min of incubation. These conditions were selected based on clinical protocols for mild hyperthermia treatment in cancer therapy [29].

As the results show, under these conditions, the modified compositions release over 65% of the encapsulated cytostatic agent, whereas the unmodified niosomes release only 35%. This again demonstrates the temperature-sensitive properties of the elaborated copolymer-modified niosomes.

Considering the release profiles at ambient temperature and under mild hyperthermia conditions, it can be concluded that the release of daunorubicin from the smart niosomes is likely controlled by a combination of diffusion and membrane destabilization processes. Temperature-dependent polymer-induced structural rearrangements may create transient pores or defects in the membrane, thereby enhancing drug diffusion. Therefore, the release mechanism is likely to be a combination of temperature-triggered membrane perturbation and passive diffusion of daunorubicin through the bilayer.

### 2.4. Storage Stability of Niosomes

Due to the colloidal instability of nanovesicular carriers such as niosomes and liposomes, we sought to evaluate the storage stability of plain or copolymer-modified niosomes by tracking changes in particle size, size distribution, and encapsulated daunorubicin content over a 30-day period at 4 °C. Based on the results presented in Table 5, both plain niosomes and those functionalized with DHP-PIPOX or DHP-PETEGA demonstrated consistent stability under these conditions. No significant variations were observed in the key formulation parameters. This stability is likely due to the presence of cholesterol and the incorporated polymer, probably through steric repulsion between the vesicles, which prevents shrinkage or fusion of the niosomes [30].

### 2.5. In Vitro Cytotoxicity Evaluation

**Figure 4:**
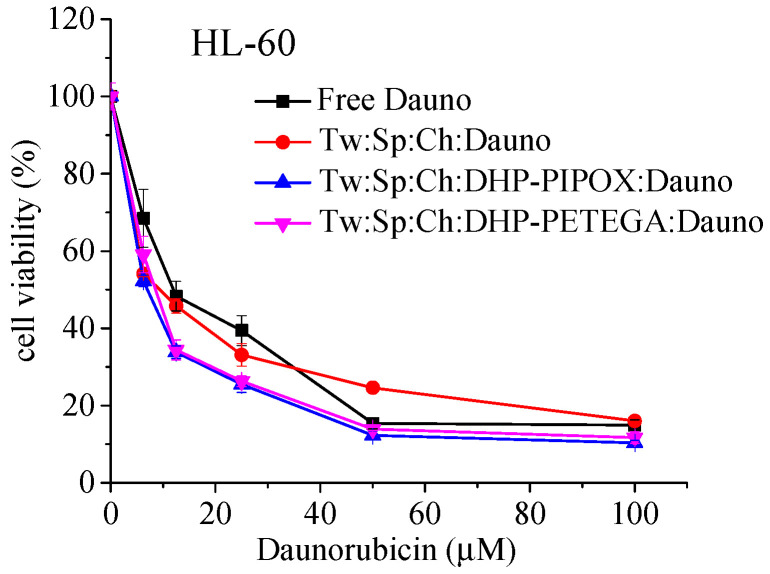
Concentration–response curves evaluated by a standard MTT test after 72 h treatment of human acute myeloid leukemia (HL-60) cell line with varying concentrations of free or nanoformulated daunorubicin hydrochloride. Each data point represents a mean value ± SD (n = 8).

The cytotoxic effect of free or niosomal daunorubicin HCl was evaluated against HL-60 cells by a standard MTT assay. The data, presented as concentration–response curves (expressed as % of untreated control), are depicted in Figure 4. The curves were processed by nonlinear regression analysis, and the calculated equi-effective concentrations IC_50_ are presented in Table 6.

The presented data clearly show that HL-60 cells are highly sensitive to daunorubicin hydrochloride (see Figure 4). After 72 h of exposure, a pronounced, concentration-dependent cytotoxic effect was observed for both the free drug and its corresponding niosomal formulations, with over 80% of tumor cells eradicated at the highest concentration tested. Additionally, niosomal daunorubicin showed a higher antiproliferative effect, as demonstrated by its lower IC_50_ values (Table 6).

**Figure 5:**
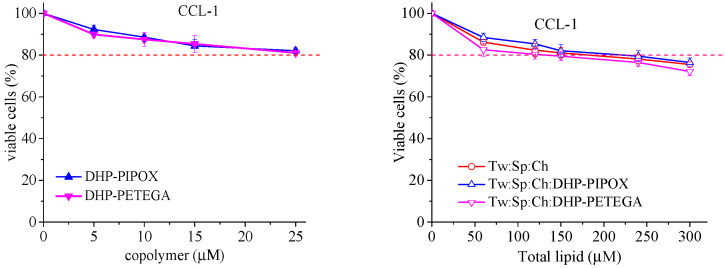
Concentration–response curves established by the MTT dye reduction assay after 72 h of continuous exposure of mouse fibroblast cells (CCL-1) to varying concentrations of copolymer solutions or non-loaded plain and copolymer-modified vesicles. Each data point represents an average arithmetic value ± standard deviation of at least 8 independent experiments.

To verify that the higher cell suppression activity of the nanoformulated agent was not due to the inherent cytotoxicity of the carriers or the polymers themselves, normal mouse fibroblasts (CCL-1 cell line) were treated with empty niosomes (either plain or modified) as well as with aqueous solutions of the modifying copolymers at concentrations similar to those in the previous experiment. The concentration–effect curves are presented in Figure 5.

The presented results clearly show that both temperature-sensitive polymers are practically devoid of cytotoxicity across the entire studied concentration range, while the empty vesicles exhibit only marginal cytotoxicity, as they do not cause more than a 25% reduction in cell viability, even at high concentrations [31].

Thus, the observed higher cytotoxic effect of niosomal daunorubicin, together with its concomitant delayed release under physiological conditions, indicates unequivocally that besides the hydrophilic corona surrounding the membranes of polymer-modified vesicles, the latter are able to interact with tumor cells, allowing immediate contact with and penetration of the incorporated cytostatic by direct exchange and/or fusion with the membranes. Given the data from the in vitro release profiles of daunorubicin HCl, in the absence of interactions between the niosomes and the cells, the latter would be exposed to only 30–50% of the free cytostatic concentrations and, accordingly, their cytotoxic effects would be marginal.

## 3. Materials and Methods

### 3.1. Materials

Daunorubicine hydrochloride, sorbitan monostearate (Span 60), polyoxyethylene sorbitan monostearate (Tween 60), cholesterol, 5(6)-carboxyfluorescein, and SpectraPor4 (cut off 12–14,000, regenerated cellulose) were purchased from Sigma-Aldrich (FOT, Sofia, Bulgaria), along with SnCl_4_ (Sigma-Aldrich, 99.995% trace metals basis), 1-Hexadecanol (HAD, ReagentPlus^®^, 99%, Sigma-Aldrich), and glycidyl hexadecyl ether (GHE, technical grade, Sigma-Aldrich). Normal mice fibroblasts (CCL-1 (CCL-1TM, NCTC clone 929) were purchased from the American Type Culture Collection (ATCC, Manassas, VA, USA) (https://www.atcc.org/ (accessed on 4 September 2025)), while the acute myelocyte leukemia-derived HL-60 cell line was obtained from the German Collection of Microorganisms and Cell Cultures (https://www.dsmz.de/ (accessed on 4 September 2025)), Braunschweig, Germany.

### 3.2. Methods

#### 3.2.1. Synthesis of Copolymers

DHP-PiPOX and DHP-PETEGA were synthesized by conjugation of pre-synthesized poly(2-isopropyl-2-oxazoline) (PiPOX) or poly(ethoxytriethylene glycol acrylate) (PETEGA) to 1,3-dihexadecyl-propane-2-ol (DHP). The lipid-mimetic moiety (DHP, M_w_ = 540 g/mol) was synthesized following a previously reported procedure [32]. Specifically, 1-hexadecanol (20 g, 0.0824 mol, 1 eq.) and glycidyl hexadecyl ether (25.85 g, 0.0866 mol, 1.05 eq.) were melted together under an argon atmosphere, followed by the addition of SnCl_4_ (0.13 mL) as a catalyst. The mixture was stirred at 115 °C for 24 h, after which a second portion of SnCl_4_ (0.13 mL) was added, and stirring was continued for an additional 70 h. The product was purified by two recrystallizations from cold hexane to afford the final compound as a white solid (yield: 22.5 g, 55%). IR (ATR, cm^−1^): ν = 3472, 2928, 2850, 1622, 1568, 1537, 1433, 1309, 1242, 1087, 892, 644. ^1^H NMR (600 MHz, CDCl_3_, δ): 3.95 (m, 1H), 3.46 (m, 8H), 1.57 (quint, 4H, J = 6.5 Hz), 1.26 (br s, 52H), 0.88 (t, 6H, J = 6 Hz).

PiPOX with an average molar mass number (M_n_) of 4300 g/mol, *Đ* = 1.18, and PETEGA with M_n_ = 4220 g/mol, *Đ* = 1.22, were synthesized by cationic ring-opening polymerization (CROP) as described by Toncheva et al., 2013, and by atom transfer radical polymerization (ATRP) according to Toncheva-Moncheva et al., 2011., respectively [24,25]. The cloud points of PiPOX and PETEGA, as well as their glass transition temperatures, determined by differential scanning calorimetry (DSC), are given in Table 2.

#### 3.2.2. Preparation of Empty and Daunorubicin-Loaded Niosomes

##### Preparation of Empty and 5(6)-Carboxyfluorescein-Loaded Niosomes

Empty conventional and copolymer-modified niosomes were prepared via the thin-film hydration method, followed by pulsatile sonication (⌀ = 2 mm, Bandelin Sonoplus HD2200, Berlin, Germany). The lipid film was formed by evaporation at 150 rpm of the chloroform solution of Span 60, Tween 60, and cholesterol (30 µmol/mL total mixture). In the case of modified vesicles, to the organic phase we also added the chloroform–methanolic solution of investigated copolymers (DHP-PiPOX or DHP-PETEGA) in concentration 0.5, 1, and 2.5 mol%. Afterward, the film was hydrated with deionized water at 50 °C for 60 min, or in case of the carboxyfluorescein-loaded niosomes, the thin lipid film was hydrated with carboxifluorescein solution (50 mM). The sonication step included 2 min of pulsatile sonication (20 s action/10 s pause) at 30% amplitude. As a subsequent step, gel filtration via Sephadex G50 columns was performed to exude the unentrapped carboxyfluorescein and to obtain final dispersions for successive analysis. Briefly, the niosomal suspensions were passed through a PD-10 column containing Sephadex G50, pre-equilibrated with water. As the niosomes were larger than the carboxifluorescein molecules, the niosomes were excluded from the gel matrix pores and eluted first, separating from the non-entrapped carboxifluorescein molecules. The purified niosomes were collected and used for further evaluation.

##### Preparation of Daunorubicin Hydrochloride-Loaded Niosomes

Daunorubicine HCl was loaded into niosomes by a remote loading procedure using the transmembrane ammonium gradient method. In brief, niosomes were prepared by hydrating the lipid film with an aqueous solution of ammonium sulfate (240 mM) and 1 mM EDTA in a round-bottomed flask at 60 °C, and the flask was rotated at 150 rpm (without vacuum). The prepared vesicles were sonicated as described above. Afterwards, the non-entrapped amount of ammonium sulfate was removed by gel filtration on PD-10 Sephadex G50 columns, pre-equilibrated with PBS buffer (pH 7.4); thus, the ammonium gradient was formed. Then aliquot part of the daunorubicin.HCl solution (4 mmol/mL) was added to an aliquot of the niosome suspension, and the resulting samples were incubated for 1 h at 50 °C. The non-entrapped drug was removed by gel filtration on Sephadex G50 columns, as described above.

### 3.3. Characterization of Niosomes

#### 3.3.1. Morphology

Images from cryogenic transmission electron microscopy (cryo-TEM) were acquired using a Tecnai F20 X TWIN microscope (FEI Company, Hillsboro, OR, USA) equipped with a field emission gun, operating at an acceleration voltage of 200 kV. The images were captured with a Gatan Rio 16 CMOS 4k camera (Gatan Inc., Pleasanton, CA, USA) and processed using Gatan Microscopy Suite (GMS) software (Gatan Inc., Pleasanton, CA, USA). Sample preparation involved vitrifying aqueous solutions on grids with holey carbon film (Quantifoil R 2/2; Quantifoil Micro Tools GmbH, Großlöbichau, Germany). Before use, the grids were treated for 15 s in oxygen plasma using a Femto plasma cleaner (Diener Electronic, Ebhausen, Germany). Cryo-samples were prepared by applying a droplet (3 μL) of the suspension onto the grid, blotting with filter paper, and immediately freezing in liquid ethane using an automated blotting device, the Vitrobot Mark IV (Thermo Fisher Scientific, Waltham, MA, USA). After preparation, the vitrified samples were stored in liquid nitrogen until they were loaded into a cryo-TEM holder Gatan 626 (Gatan Inc., Pleasanton, CA, USA) and examined in the TEM at −178 °C.

#### 3.3.2. DLS Analysis

The size, size distribution pattern, and zeta potential of the obtained nanocarriers were evaluated by a Zetasizer NanoZS (Malvern Instruments, Malvern, UK), operated with a 633 nm laser. The experiment was performed in triplicate at a scattering angle of 175 °C and at ambient (25 °C) and elevated (40 °C) temperatures for the conventional and copolymer-modified niosomes. The results are presented as mean values ± SD (n = 3).

#### 3.3.3. Entrapment Efficacy (EE)

The entrapment efficacy of daunorubicine HCl was determined by UV-VIS spectroscopy (λ = 494, r^2^ = 0.99903) as follows: a sample of niosomal dispersion (200 μL) was dissolved in isopropanol (200 μL) and diluted with water to 1 mL and analyzed. The same procedure was used to determine the daunodubicine HCl in non-purified niosomes. The entrapment efficacy was calculated as follows:

(1) $$EE\left(\%\right)=\frac{Dnio}{Dtot}\times 100$$

where *Dnio* is the amount of drug loaded into the niosomes (after gel filtration), and *Dtot* is the total amount of drug in the niosomal suspension (before gel filtration)

### 3.4. Evaluation of Thermal Responsiveness of Niosomes

To evaluate whether the incorporation of hydrophobically modified PiPOX and PETEGA copolymers can induce temperature-dependent triggered release, 100 μL of CF-loaded niosomes were diluted to 2 mL with PBS (pH 7.4) and the samples were incubated for 40 min at ambient temperature and at 42 °C in a thermostatic water bath. At the beginning and at the end of the incubation period, the fluorescence of the samples was measured at λemm = 520 nm and λex = 490 nm via a Hitachi 7000 fluorescence spectrophotometer. To determine the total intensity, niosomes were disrupted by adding 100 μL of 10% *v*/*v* solution of Triton X100, and carboxyfluorescein leakage was calculated applying the following equation:

(2) $$\mathrm{CF leakage}\left(\%\right)=\frac{In}{Itot}\times 100$$

where *In* is the fluorescence intensity at time (n), and *Itot* is the total intensity after disruption by the niosomes.

### 3.5. In Vitro Daunorubicin Release

The release experiments were performed by the dialysis method at three temperatures (25, 37, and 42 °C). In brief, a 1 mL niosomal suspension (corresponding to 1.4 mg/mL daunorubicin) or a 1 mL aqueous daunorubicin solution (at the same concentration) were placed in dialysis bags (SpecrtaPor, MWCO = 12–14 kDa, Sigma-Aldrich, Steinheim, Germany). The bags were immersed in 50 mL PBS in a thermostatic water bath. At predetermined time intervals, 2 mL of the acceptor phase was withdrawn for analysis and immediately replaced with 2 mL of fresh buffer. The absorbance of the samples was measured at 494 nm using a UV-Vis spectrophotometer, and the drug concentration was calculated according to a pre-built standard curve (linear in the range of 2 μg to 80 μg, correlation coefficient R^2^ = 0.99903). The results are presented as the cumulative release (%).

### 3.6. Stability Evaluation

The storage stability of the optimal niosomal formulations was evaluated by storing the samples for 30 days at refrigerator temperature (4 ± 2 °C) and analyzing, from a comparative viewpoint, any alterations in their properties, such as their size, PDI, zeta potential, and EE values, at the beginning and the end of the tested period.

### 3.7. Assessment of Cytotoxicity of Daunorubicin-Loaded Conventional and Copolymer-Modified Thermoresponsive Niosomes

#### 3.7.1. Cell Lines and Culture Conditions

The in vitro antiproliferative effects of free or encapsulated daunorubicin hydrochloride were examined against the acute myelocyte leukemia-derived HL-60 cell line (German Collection of Microorganisms and Cell Cultures (https://www.dsmz.de/ (accessed on 4 September 2025))), while the cytotoxic potential of the copolymers were evaluated on normal mice fibroblasts (ccl-1) (CCL-1TM, NCTC clone 929, American Type Culture Collection—ATCC, Manassas, VA, USA) (https://www.atcc.org/ (accessed on 4 September 2025)). HL-60 cells were cultured in RPMI 1640 medium supplemented with 10% fetal bovine serum (FBS) and 5% L-glutamine, maintained at 37 °C and under 5% humidified CO_2_. CCL-1 cells were maintained following the recommendations of ISO 10993-5, Annex C (ISO 10993-5:2009 2017) [31]. CCL-1 cells were grown in Eagle’s Minimum Essential Medium (MEM-A, Capricorn^®^, Munich, Germany), enriched with 10% heat-inactivated horse serum (HOS-1A, Capricorn^®^, Munich, Germany) and 2 mM L-glutamine (G7513, Sigma-Aldrich, Steinheim, Germany), under identical culture conditions to those used for the HL-60 cells.

#### 3.7.2. MTT Colorimetric Assay

The in vitro antiproliferative activity of daunorubicin in the niosomal formulations and free drug was assessed using an established method for evaluating cell viability, namely the Mosmann MTT dye reduction assay, with minor modifications [33]. Exponentially growing cells were harvested and seeded into 96-well plates (100 μL/well) at appropriate densities (1 × 105 for suspension cultures). After 24 h of incubation, cells were treated with five distinct concentrations of the nanocarriers based on daunorubicin content (6.25, 12.5, 20, 54.2, and 100 μM). Following a 72 h exposure period, 10 μL/well of filter-sterilized MTT substrate solution (10 mg/mL in PBS) was added to each well. An additional 2–4 h of incubation facilitated the formation of insoluble purple formazan crystals. These crystals were then solubilized by the addition of 100 μL/well isopropyl alcohol solution containing 5% formic acid, before measuring absorbance at 580 nm. Absorbance readings were corrected against MTT–isopropanol solution. Cell survival rates were determined as a percentage relative to the untreated control group (100% cell viability). Additionally, IC_50_ values were derived from the concentration–response curves.

### 3.8. Statistical Analysis

Statistical analysis was performed using Excel via one-way analysis of variance (ANOVA.) The value of *p* < 0.05 was denoted as statistically significant.

## 4. Conclusions

In this study, we successfully developed thermosensitive niosomal formulations, by incorporation of the temperature-sensitive copolymers DHP-PiPOX and DHP-PETEGA, for the targeted delivery of daunorubicin hydrochloride. The nanovesicles exhibited suitable physicochemical properties, specifically sizes and size distributions within the optimal range for systemic administration (below 200 nm) and low polydispersity indices. Notably, the niosomes demonstrated stability under physiological conditions, minimizing premature drug leakage. Importantly, they were capable of actively releasing daunorubicin in response to mild hyperthermia (42 °C), highlighting their potential for temperature-triggered drug delivery in cancer therapy. These findings support the further investigation of thermosensitive niosomes as promising carriers for controlled, site-specific delivery of chemotherapeutic agents. However, future investigations exploring in vivo performance and behavior in complex biological systems could provide additional insights into their potential as thermoresponsive drug delivery platforms with clinical relevance.

## Figures and Tables

**Table 1:** Physicochemical properties of ultrasound-treated plain (Tw60:Sp60:Ch 3.5:3.5:3 molar ratio) niosomes and their copolymer-modified counterparts.

Sample Code	Polymer (mol%)	D_h_ (nm) ± SD		PDI ± SD		ζ-Potential (mV) ± SD	
		25 °C	40 °C	25 °C	40 °C	25 °C	40 °C
S1	-	133 ± 1.7	142 ± 10.7	0.3 ± 0.09	0.46 ± 0.05	−11.2 ± 1.5	−11.0 ± 2.1
DHP-PiPOX-modified niosomes							
S2	0.5	100 ± 2.5	115 ± 9.5	0.24 ± 0.06	0.24 ± 0.02	−26.1 ± 0.07	−18.5 ± 0.5
S3	1	110 ± 6.9	126 ± 5.2	0.34 ± 0.08	0.27 ± 0.01	−44.1 ± 0.35	−42.2 ± 0.6
S4	2.5	112 ± 5.3	136 ± 8.5	0.24 ± 0.05	0.31 ± 0.09	−26.1 ± 0.01	−19.2 ± 1.4
DHP-PETEGA-modified niosomes							
S5	0.5	94 ± 8.5	76.2 ± 9.7	0.32± 0.05	0.19± 0.02	−12.8 ± 2.6	−12.5 ± 0.4
S6	1	100 ± 9.7	117 ± 15.6	0.28± 0.06	0.17 ± 0.2	−15.1 ± 0.23	−13.5 ± 0.35
S7	2.5	119 ± 7.6	163 ± 4.2	0.27 ± 0.02	0.17 ± 0.05	−12.5 ± 0.14	−10.2 ± 0.78

**Table 3:** Daunorubicin hydrochloride encapsulation efficacy of niosomes after passive and active drug loading.

Sample Code	Encapsulation Efficacy (%)	
	Passive Loading	Active Loading
Tw60:Sp60:Ch:Dauno (sN1)	18.9 ± 3.4	71 ± 2.8
Tw60:Sp60:Ch:DHP-PIPOX:Dauno (sN4)	16.8 ± 6.2	68.6 ± 4.1
Tw60:Sp60:Ch:DHP-PETEGA:Dauno (sN7)	16.2 ± 4.3	66.5 ± 3.2

**Table 4:** Size, size distribution, and zeta potential of loaded plain and polymer-modified thermosensitive niosomes at 25 °C. Values represent means ± S.D. (n = 3).

Composition	Copolymer (2.5 mol%)	Dauno:Surfactants (mol:mol)	D_h_ (nm) ± SD	PDI ± SD	ζ-Potential (mV) ± SD
sN1	-	2:15	157 ± 2.3	0.29 ± 0.05	−10 ± 1.8
sN4	DHP-PiPOX	2:15	155 ± 5.9	0.25 ± 0.04	−22 ± 2.4
sN7	DHP-PETEGA	2:15	158 ± 4.8	0.29 ± 0.02	−11 ± 2.5

**Table 5:** Storage stability of optimal daunorubicin-loaded niosomes after one month of storage at 4 ± 2 °C.

Sample		Size (nm)	PDI	ζ Potential (mV)	EE (%)
(sN1)	Initial	157 ± 2.2	0.29 ± 0.05	−10 ±1.8	71 ± 2.8
	After 1 month storage	161 ± 2.2	0.31 ± 2.2	−10.8 ± 3.4	66 ± 1.6
(sN4)	Initial	155 ± 5.9	0.25 ± 0.04	−22 ± 2.4	68.6 ± 4.1
	After 1 month storage	151 ± 7.5	0.29 ± 0.04	−24 ± 3.8	66.7 ± 2.2
(sN7)	Initial	158 ± 4.8	0.29 ± 0.02	−11 ± 2.5	66.5 ± 3.2
	After 1 month storage	162 ± 5.4	0.32 ± 0.06	−10.9 ± 2.2	62.1 ± 4.2

**Table 6:** Equi-effective concentrations (IC_50_) of niosomal and free daunorubicin against human tumor cell line HL-60.

Sample	HL-60
Free daunorubicin	12.14 ± 2.15
sN1	9.85 ± 3.01
sN4	6.91 ± 2.42
sN7	8.54 ± 1.18

## Data Availability

The original contributions presented in this study are included in the article. Further inquiries can be directed to the corresponding author.

## Abbreviations

The following abbreviations are used in this manuscript:

DHP-PiPOX	1,3-dihexadecyl-propane-2-ol-poly(2-isopropyl-2-oxazoline
DHP-PETEGA	1,3-dihexadecyl-propane-2-ol-poly(ethoxytriethylene glycol acrylate
HL-60	acute myelocyte leukemia-derived cells
CCL-1	normal mouse fibroblasts
Cryo-TEM	cryogenic transmission electron microscopy
DLS	dynamic light scattering
MTT	3-(4,5-dimethylthiazol-2-yl)-2,5-diphenyltetrazolium bromide
Tw60	polyoxyethylene sorbitan monostearate
Sp60	sorbitan monostearate
Ch	cholesterol
